# Spontaneous regression of metastatic disease after palliative debulking surgery for heavily pre-treated extraskeletal myxoid chondrosarcoma: A case report

**DOI:** 10.1177/20363613251392654

**Published:** 2025-11-27

**Authors:** Hailey Kathryn Carroll, Anna Keogh, Simon Barry, Aurelie Fabre, Gary O’Toole, John Crown, Deirdre O’Mahony, Kenneth Feeley, Asif Muneer, Richard M. Bambury

**Affiliations:** 1 Department of Medical Oncology, 57983 Cork University Hospital, Cork, Ireland; 2 Department of Medical Oncology, 58038 St Vincent’s University Hospital, Dublin, Ireland; 3 University College Dublin School of Medicine Graduate Studies, 37438 University College Dublin, Dublin, Ireland; 4 Department of Pathology, 58038 St Vincent’s University Hospital, Dublin, Ireland; 5 Department of Orthopaedic Surgery, 58038 St Vincent’s University Hospital, Dublin, Ireland; 6 Department of Orthopaedic Surgery, 11358 National Orthopaedic Hospital Cappagh, Dublin, Ireland; 7 Department of Medical Oncology, St Vincent’s Private Hospital, Dublin, Ireland; 8 Department of Medical Oncology, Bon Secours Hospital, Cork, Ireland; 9 Department of Pathology, Bon Secours Hospital, Tralee, Ireland; 10 Male Genital Cancer Centre, Department of Urology, 8964 University College London Hospitals NHS Foundation Trust, London, UK; 11 National Institute for Health and Care Research (NIHR) Biomedical Research Centre, 8964 University College London Hospitals NHS Foundation Trust, London, UK; 12 Cancer Research @UCC, 8795 University College Cork, Cork, Ireland

**Keywords:** extraskeletal myxoid chondrosarcoma, spontaneous regression, lung metastases, sarcoma, debulking surgery

## Abstract

Spontaneous regression of metastatic disease in the setting of advanced cancer is a poorly understood clinical phenomenon which occurs infrequently across all tumour types but exceptionally rarely in soft tissue sarcomas. Extraskeletal myxoid chondrosarcoma (EMC) is a rare subtype of soft tissue sarcoma that is poorly responsive to systemic treatment, providing limited options to patients with metastatic disease. We report spontaneous regression of lung metastases in a patient with EMC after re-resection of the primary tumour, which was performed with palliative intent for symptom control after multiple lines of systemic treatment. The patient has remained disease-free and is now more than 5 years post-surgery. To our knowledge, this is the first described case of spontaneous regression of metastatic disease following resection of a primary tumour in a patient with EMC.

## Introduction

Extraskeletal myxoid chondrosarcoma (EMC) is an exceedingly rare sarcoma that most commonly originates in the soft tissues of the extremities.^1^ In patients with localised disease, surgical resection is the primary approach to management.^2^ While EMCs often follow an indolent clinical course, local and distant recurrence occurs in over 40% of patients.^3,4^ EMCs are classically insensitive to cytotoxic treatments, which leads to difficulty in providing meaningful systemic therapy for patients with metastatic disease. We present a case of spontaneous regression of metastatic disease after palliative intent resection of a primary pelvic EMC in a patient who had received multiple lines of systemic therapy.

## Case description

**Image 1.**
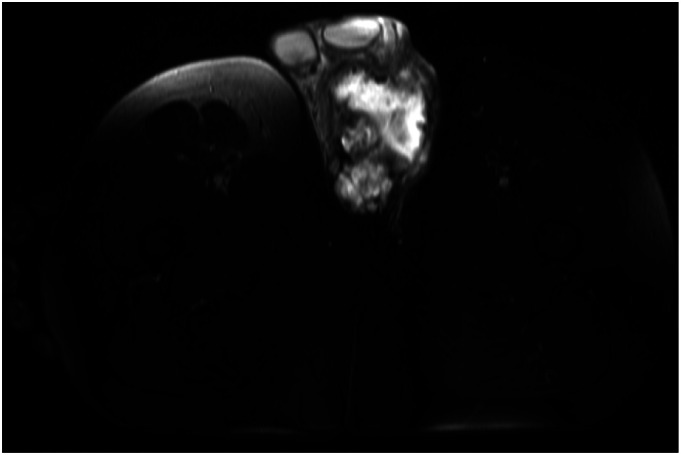
MRI showing extraskeletal myxoid chondrosarcoma in the left left groin, lateral to the left testis.

**Image 2.**
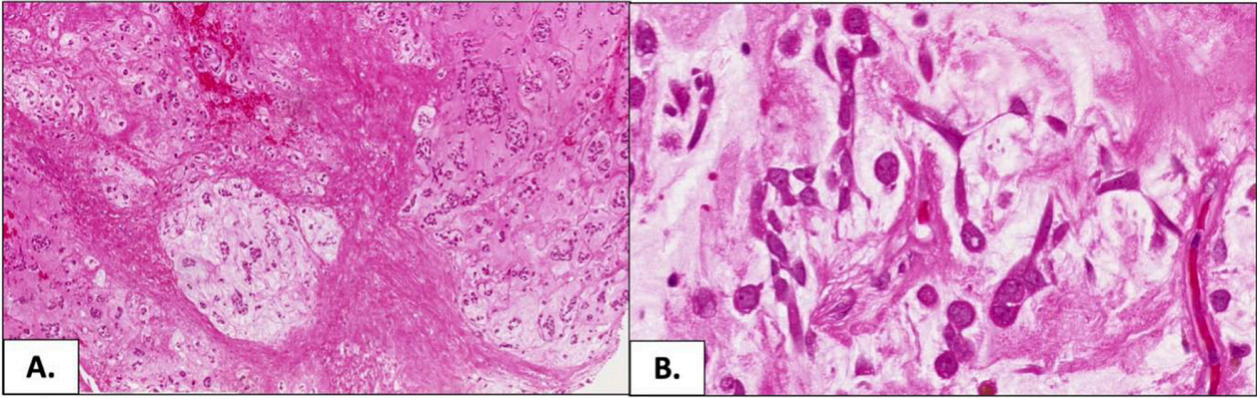
Low and high-power images of the tumour showing classic histological features of extraskeletal chondromyxoid sarcoma. (a) A low-power image shows a nodular architecture containing bland cells with eosinophilic cytoplasm lying within a myxoid stroma (magnification 4X). (b) A high-power image further demonstrates the myxoid stroma. The tumour cells are uniform and form interconnecting cords with long, delicate processes (magnification 40X).

In March 2015, a 56-year-old male with no medical history presented with a 4-month history of an enlarging, palpable mass in his left groin. Ultrasound and magnetic resonance imaging (MRI) confirmed a 5.5 cm mass lateral to the left testis (Image 1). A biopsy was performed at a local hospital, which revealed extraskeletal myxoid chondrosarcoma. Computed tomography of the thorax, abdomen, and pelvis (CT TAP) was performed, and no other disease sites were identified. The patient was referred to a large tertiary cancer centre and subsequently underwent an en-bloc resection of the left scrotal mass. Histology confirmed a low-grade myxoid extraskeletal chondrosarcoma, measuring 6 cm in maximal dimension. There was no evidence of dedifferentiation or lymphovascular invasion, and the mitotic count was <1 per 10 high-powered fields (HPF), and no lymphovascular invasion seen (Image 2). The microscopic surgical margins involving the left ischium, urethra, and left scrotum were positive for tumour cells (R1 resection). The histologic diagnosis was subsequently confirmed molecularly by fluorescence in situ hybridisation (FISH), demonstrating NR4A3 gene rearrangement. He recovered well and did not receive adjuvant therapy. He subsequently proceeded to a period of active surveillance.

In March 2016, surveillance magnetic resonance imaging (MRI) identified a 2.5 cm area of lobulated tissue in the left perineum consistent with local disease recurrence. The patient was commenced on first-line sunitinib, an oral multi-targeted tyrosine kinase inhibitor. He experienced grade 1 gastrointestinal toxicity and hair depigmentation. After 6 months of treatment, a follow-up MRI of the pelvis measured the lobulated mass within the left side of the perineum at approximately 4.1 cm, consistent with local disease progression. At this stage, he was referred for consideration of further surgery, but ultimately, this did not proceed due to the anticipated morbidity of the procedure.

Between August and November 2017, he received five cycles of the PD-L1 inhibitor atezolizumab on a basket clinical trial. He had grade 2 immune-related thyroiditis during his treatment. After 3 months of treatment, subsequent scans demonstrated further local disease progression. An MRI of the pelvis performed in November 2017 measured the mass at 11.9 cm, and CT TAP remained negative for metastatic disease.

In February 2018, he began third-line treatment with trabectedin. He tolerated this well and experienced minimal toxicity. An updated CT TAP was performed in May 2018 and identified multiple new, bilateral, sub-centimetre pulmonary nodules suspicious for metastases. He continued trabectedin until imaging in August 2018 confirmed further progressive disease in the pelvis with the persistent large lobulated mass in the perineum now measuring 15.8 cm in longest diameter. The mass was now encasing the root of the penis, with direct invasion into the anterior aspect of the left adductor group muscles in the left groin. The CT TAP showed stable pulmonary metastases and no other metastatic disease.

At this stage, his case was discussed at the National Sarcoma Multidisciplinary Team Meeting (MDT). Due to the anticipated morbidity, particularly in the setting of multiple bilateral lung metastases, radical pelvic surgery was again deemed inappropriate. He was referred for consideration of palliative radiotherapy, which he subsequently declined. Subsequently, next-generation sequencing (NGS) using the ONCOmine platform was performed in April 2019. It showed no actionable genetic alterations, and a decision was made not to pursue further systemic therapy due to the previously demonstrated treatment-resistant nature of this disease.

**Image 3.**
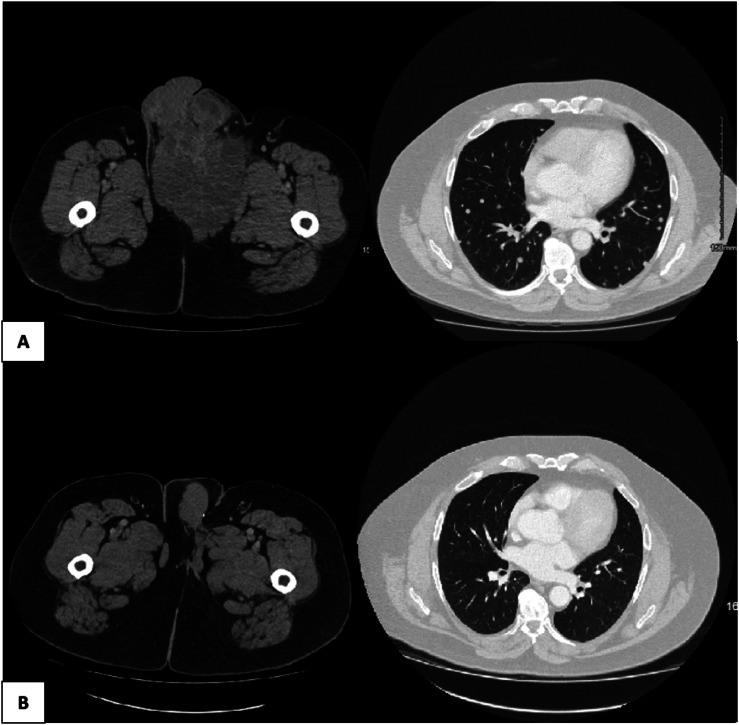
Cross-sectional imaging of primary tumour and metastatic disease in August 2019, before palliative intent debulking surgery (a) and (b) 5 years post-surgery in November 2024.

The patient continued to experience worsening pelvic pain and pressure symptoms related to his primary tumour. By August 2019, imaging demonstrated an enlarging perineal mass, now measuring 18 cm, and enlarging bilateral lung metastases (Image 3(a)). Due to his worsening symptoms, palliative debulking surgery was reconsidered, and he was referred for review in the United Kingdom at the University College London Hospitals.

**Image 4.**
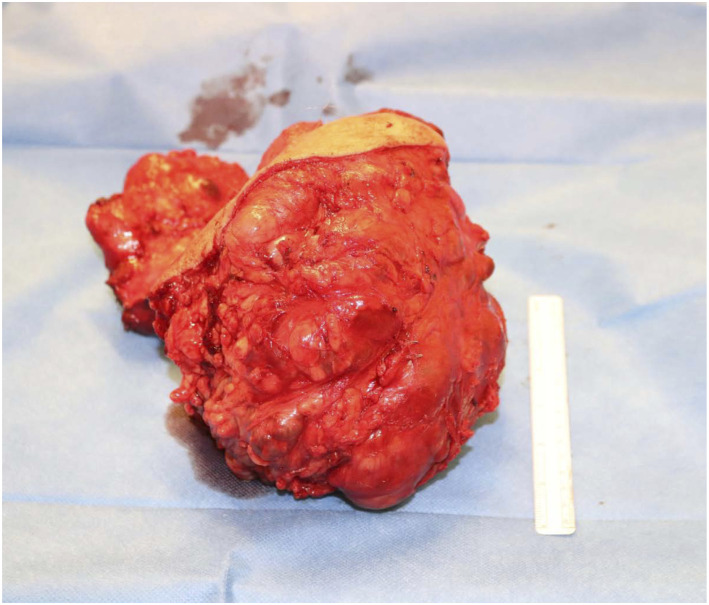
Intraoperative photograph of resected extraskeletal myxoid chondrosarcoma.

Four and a half years after his initial diagnosis, the patient underwent debulking of his primary pelvic tumour in October 2019. An excision of the left abdominoperineal area, thigh, left crural and corporal tissue was performed. The resected tumour was lobulated and myxoid with a maximum dimension of 18 cm (Image 4). On microscopic examination, extensive areas of necrosis were identified. Infiltration of the fibroadipose tissue and focal entrapment of the vas deferens structures were noted. Microscopically, the tumour was composed of modules of spindle cells with mild nuclear atypia. There were occasional mitoses and large areas of haemorrhage and necrosis. Vascular invasion was present. The pathology showed a grade 2 extraskeletal myxoid chondrosarcoma, with focally positive surgical margins.

After his surgery, surveillance with cross-sectional imaging was resumed. In January 2020, a CT TAP showed a decrease in the size of his bilateral pulmonary metastases. By May 2020, imaging noted full resolution of all previously described metastatic pulmonary nodules. The most recent cross-sectional imaging performed in November 2024 demonstrates no evidence of disease (Image 3(b)).

## Discussion

Extraskeletal myxoid chondrosarcoma (EMC) is an ultra-rare, soft tissue sarcoma driven by chromosomal translocations involving the gene NR4A3. This rare subtype of soft tissue sarcoma accounts for approximately 1% of all soft tissue sarcomas.^5^ Despite their generally indolent behaviour, EMCs have a high rate of recurrence. Unfortunately, for patients with recurrent or metastatic disease not amenable to resection, there is a lack of evidence surrounding beneficial systemic treatment, with the use of chemotherapy not conferring an overall survival benefit.^5,6^ One of the most extensively published case series of metastatic EMC observed no significant response to systemic treatment in 21 patients.^7^ A retrospective review using SEER data of 270 EMC cases also demonstrated that chemotherapy and radiotherapy did not improve overall survival, which is currently estimated to be between 77%–89% at 5 years.^5^ The lack of response to systemic treatment in metastatic disease emphasises the importance of aggressive locoregional oncological resection as the cornerstone of therapy for patients with locoregional disease and significantly correlates with overall survival.^6^

However, despite the recognised importance of robust surgical resection in the curative setting, debulking surgery is not the standard of care treatment for metastatic sarcoma. Palliative-intent surgery carries significant risk and morbidity with limited evidence of survival benefit.^8^ Additionally, accumulating pre-clinical and clinical evidence suggests the physiological stress of surgery may accelerate the progression of metastatic disease by surgery-induced immunosuppression, growth factor and proangiogenic compound release, and tumour cell dissemination.^9,10^ Therefore, the decision to perform debulking surgery in patients with advanced disease should be made by a multidisciplinary sarcoma team at a large-volume reference centre, with careful consideration of the individual patient’s clinical features, symptom burden, treatment history, performance status, overall prognosis, and both the expected and potential complications associated with surgery.

Spontaneous regression of cancer is a rare yet well-documented phenomenon. Despite being first defined in the literature in 1956 by Drs. Warren Cole and Tilden Everson, spontaneous regression has been described in plants, animals, and humans for hundreds of years.^11,12^ It is defined as the partial or complete disappearance of an untreated or sub-optimally treated malignant tumour and occurs approximately one in every 60,000-100,000 cases across all cancer types.^13–15^ It is most commonly described in renal cell carcinomas, neuroblastomas, malignant melanomas, and lymphomas.^15,16^ Cases of spontaneous regression in sarcoma are not well described in the literature, with less than 20 cases reported in the English-language literature since 1946 across all subtypes.^13,14,17^

The mechanism of spontaneous regression in cancer is not well understood and remains perplexing to the science and medical communities. Studies suggest this is likely due to a complex interplay of multiple factors, which lead to a cytotoxic immune response against the tumour.^18^ Case reports of spontaneous regression have been reported after febrile episodes, infections or vaccinations, surgery or trauma, and blood transfusions. In addition to the immune system, other elements or processes that may play a role in spontaneous regression include the tumour microenvironment, epigenetic modifications, hormones, and other physiological factors. Still, ultimately, the mechanism has not been elucidated.^18–20^

Surgical intervention has been suggested to enhance the immunological resistance to tumour growth and may potentiate a host immune response on remaining cancer cells.^18,21^ This patient’s case is unique as the spontaneous regression occurred after multiple lines of therapy and interventions and several years following his initial cancer diagnosis. A complete regression of the pulmonary metastases was observed approximately 7 months after his surgery. We hypothesise that the procedure induced an immune response leading to apoptotic cell death in the lung metastases. Of note, the patient had received a PD-L1 inhibitor 2 years before his surgery, which may have theoretically primed the immune system for such a result.

To our knowledge, this is the second case described in the literature of spontaneous regression of metastatic extraskeletal myxoid chondrosarcoma (EMC), but the first in a patient with a long-standing diagnosis after multiple lines of therapy. Kinoshita et al. described a case of spontaneous regression of EMC after needle biopsy in a 25-year-old with an inguinal EMC with lung metastases.^17^ However, this occurred during the initial diagnosis versus after years of treatment, such as in our patient.^17^ While case reports of rare events do not, by the nature of their existence, describe events that should be expected in treating similar patients, it is crucial to describe rare occurrences as it drives innovation and allows critical thinking around fundamental cancer pathophysiology, immune mechanisms, and therapeutic paradigms. Ultimately, understanding the immunological mechanisms of spontaneous regression of cancers would be invaluable to all stakeholders in cancer care and may shed light on predictors of treatment response, tumour behaviour, and patient outcomes.

## Conclusion

This case represents an extraordinarily rare occurrence of spontaneous regression of pulmonary metastatic disease following debulking surgery in a patient with systemic treatment-refractory metastatic EMC. While the mechanism of spontaneous regression remains unclear, this case challenges our current understanding of the natural history of EMC and raises important questions about how surgical intervention and prior treatments, including previous immunotherapy exposure, may modulate immune response. It also highlights the importance of individualised treatment decisions made by experienced multidisciplinary teams. Documentation and description of such rare phenomena in ultra-rare cancers such as EMC are essential to advance our understanding and close the knowledge gaps in tumour biology, which, in the future, may inform research strategies and therapeutic approaches for patients with limited treatment options.

## Data Availability

All data relevant to the case report is included in the article. No additional data are available.*
